# Impaired sequence generation: a preliminary comparison between high functioning autistic and neurotypical adults

**DOI:** 10.3389/fnbeh.2022.946482

**Published:** 2022-09-06

**Authors:** Elien Heleven, Tom Bylemans, Qianying Ma, Chris Baeken, Kris Baetens

**Affiliations:** ^1^Brain Body and Cognition, Department of Psychology, and Center for Neuroscience, Vrije Universiteit Brussel, Brussels, Belgium; ^2^Department of Head and Skin (UZGent), Ghent Experimental Psychiatry (GHEP) Lab, Ghent University, Ghent, Belgium

**Keywords:** social action sequencing, autism, picture sequencing task, verbal sequencing task, social cognition, mentalizing, cerebellum

## Abstract

Earlier research demonstrated robust cerebellar involvement in sequencing, including high-level social information sequencing that requires mental state attributions, termed mentalizing. Earlier research also found cerebellar deficiencies in autism spectrum disorders (ASD) which are characterized by social difficulties. However, studies on high-level social sequencing functionality by persons with ASD are almost non-existent. In this study, we, therefore, perform a comparison between behavioral performances of high-functioning ASD and neurotypical participants on the Picture and Verbal Sequencing Tasks. In these tasks, participants are requested to put separate events (depicted in cartoon-like pictures or behavioral sentences, respectively) in their correct chronological order. To do so, some of these events require understanding of high-level social beliefs, of social routines (i.e., scripts), or nonsocial mechanical functionality. As expected, on the Picture Sequencing task, we observed longer response times for persons with ASD (in comparison with neurotypical controls) when ordering sequences requiring an understanding of social beliefs and social scripts, but not when ordering nonsocial mechanical events. This confirms our hypotheses that social sequence processing is impaired in ASD. The verbal version of this task did not reveal differences between groups. Our results are the first step toward new theoretical insights for social impairments of persons with ASD. They highlight the importance of taking into account sequence processing, and indirectly the cerebellum when investigating ASD difficulties.

## Introduction

The success of everything we do in our life strongly depends on structuring our actions or thoughts in the correct order. Whether it is bringing a spoon full of soup to our mouth or participating in a political debate the order in which we sequence our motor or mental actions will lead to a delicious flavor of soup or political success, or a stain on our blouse or ego. Neuroscientists have demonstrated robust cerebellar involvement in the processing, monitoring, and production of all types of action sequences, including motor, cognitive, and social ones (Van Overwalle et al., 2020a). According to the “sequence detection” hypothesis, the cerebellum automatizes and fine-tunes all motor and mental sequences of actions *via* the signalization of prediction errors when an action does not seem to lead to its intended goal (Leggio and Molinari, 2015). As a result, the cerebellum facilitates the automatization of repeated actions so that they become predictable and smooth, and allows rapid adjustment of actions that lead to unexpected or undesirable outcomes.

Most research to date on the sequencing function of the cerebellum involved motor and cognitive processes implicating the self, and less the understanding of others, their actions, and their mental states, i.e., social mentalizing. Only recently, meta-analyses revealed a mentalizing system in the posterior cerebellar Crus 1 and 2 and strong cerebello-cortical connectivity between the posterior cerebellum and mentalizing areas in the cortex (Buckner et al., 2011; Van Overwalle et al., 2015, 2019a, 2020b, c). Novel functional magnetic resonance imaging (fMRI) studies on neurotypical participants demonstrated the involvement of the cerebellar Crus when ordering events in chronological or memorized sequences required the attribution of mental states such as beliefs (Heleven et al., 2019; Ma et al., 2021), traits (Pu et al., 2020), intentions during goal-directed navigation (Li et al., 2021), and social action prediction (Haihambo et al., 2021). In sum, these studies provide evidence for cerebellar involvement in high-level social sequence processing.

However, previous studies on mentalizing during sequencing mainly focused on neurotypical persons. While these efforts are valuable for theoretical insight, investigating clinical populations is of primordial importance since it might lead to new explanations for problems related to social functioning and potential diagnostic tools. In addition, Van Overwalle et al. (2021) argued that a systematic investigation of social sequencing performance in clinical populations might lead to a coherent theory that links a wide variety of clinical pathologies to social mentalizing impairments and posterior cerebellar deficits such as autism spectrum disorder (ASD; D’Mello et al., 2015; Olivito et al., 2018; Velikonja et al., 2019), depression (Bora and Berk, 2016; Schutter, 2016), obsessive-compulsive disorder (Narayanaswamy et al., 2016; Xu et al., 2019; Zhang et al., 2019; Jansen et al., 2020), and addiction (Maurage et al., 2011; Kornreich et al., 2013; Miquel et al., 2016; Onuoha et al., 2016). Mentalizing related to the posterior cerebellum might play a critical role in the onset, maintenance, or modulation of these psychological disorders (Van Overwalle et al., 2021).

The current study examines behavior that relies on the cerebellar sequencing function and, therefore, indirectly investigates mechanisms underlying cerebellar social functions and dysfunctions. We chose to focus on ASD since it is the clinical population with perhaps the most widely known social impairment (Velikonja et al., 2019) and which also shows systematic deficiencies in the posterior cerebellum (Wang et al., 2014; D’Mello et al., 2015). In addition, an increased risk for ASD is associated with cerebellar damage and dysfunction (Fatemi et al., 2012; Mapelli et al., 2022). This new approach to investigate sequential social processing in ASD might give additional insight into the difficulties ASD persons experience and may so complement traditional social models of ASD. Previous efforts studying sequence processing in ASD only tested children or adolescents on relatively simple social tasks, and never included social sequences involving higher-level mentalizing (Zalla et al., 2006).

**Figure 1:**
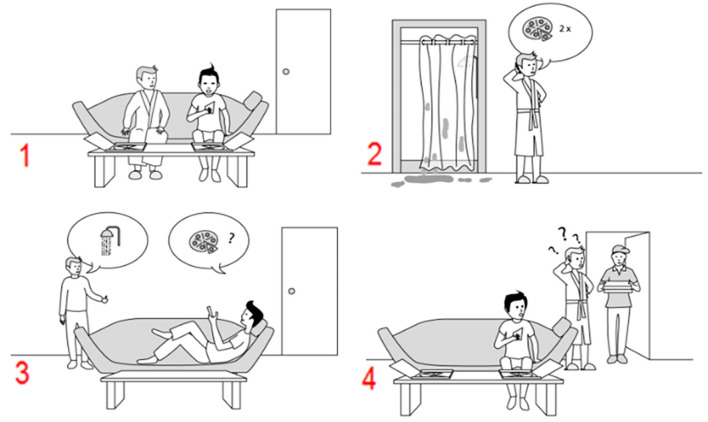
An example of a false belief sequence in the picture sequencing task (the correct order is 3-2-1-4). Participants have to select, in the correct order, the first picture on the screen, then the second picture, and so on. After each selection, the pictures move in the order indicated by the participant (Heleven et al., 2019).

Therefore, this study investigated social and non-social action sequence generation in high functioning autistic adults and compared their performance with neurotypical adults. To measure high-level mentalizing, we used an extended version of the picture and verbal sequencing tasks (Heleven et al., 2019; Van Overwalle et al., 2019b). These tasks require participants to generate the correct chronological order of a series of event sequences, depicted in pictures, or sentences. The events involve routine social or non-social sequences, and more importantly, social sequences that require the correct understanding of another agent’s mental state (for example see Figure 1). Specifically, they require the participant to make true or false belief attributions, that is, the notion that an agent’s knowledge about reality is correct (true belief) or wrong (false belief; Baron-Cohen et al., 1999). fMRI research demonstrated that both tasks recruit the cerebellum (Heleven et al., 2019) and non-invasive neurostimulation targeting the cerebellum increases task performance for neurotypical individuals (Heleven et al., 2021). In addition, cerebellar patients as compared to neurotypical persons showed impaired performance on the picture version of this task for generating the correct sequence of beliefs, but not for mechanical events or overlearned social scripts (Van Overwalle et al., 2019b).

Since sequencing is associated with the functioning of the posterior cerebellum (Van Overwalle et al., 2019c) and ASD is associated with deficits in this region (D’Mello et al., 2015; Olivito et al., 2018; Velikonja et al., 2019), we expected behavioral impairments in action sequence generation for persons with ASD as compared to neurotypical participants. We expected these differences to be most pronounced for social sequences involving mentalizing, since ASD is particularly linked to problems in social understanding and interaction (Howlin and Moss, 2012), and perhaps even for social routines, since autistic people often have reduced generalized knowledge of what happens in everyday social situations (Loth et al., 2008). Note that high-functioning autistic adults often use compensatory strategies (Livingston et al., 2020) and can successfully perform false-belief tasks (Channon et al., 2014; Eddy, 2019). Therefore, we expected ceiling effects for the accuracies of all scenarios. However, given that such strategies are often inefficient and time-consuming (Livingston and Happé, 2017), we did expect longer response times for persons with ASD in comparison with neurotypical participants.

## Methods

### Participants

In total 27 persons participated in this study. Of these, 14 were officially diagnosed with ASD by independent multidisciplinary teams at several diagnostic centers based in Belgium. Among others, the diagnostic procedure always included an extensive developmental case history based on information of the client and close others (parents and/or partner), intelligence tests, the ADOS-2, and/or ADI-R, a DSM-V interview, and tasks that measure mentalizing. This ASD group consisted of eight women and six men with an age range of 21–33 years (*M* = 24.92, *SD* = 3.80). These were all high-functioning autistic adults (i.e., with an average to high intelligence level) and were free of concurrent neurological diagnoses or comorbid psychotic disorders. We accepted other comorbid psychiatric disorders, as such comorbidities are difficult to exclude from an autistic sample (Damiano et al., 2014). Three persons reported depressive symptoms and one of these three received medication (duloextine, rilatine, and aripiprazole). Another person received medication for experienced anxiety (venlafaxine and deanxit). One person reported suffering from burnout. All others did not report any comorbid or secondary disorders or use of medication. All persons with ASD had an average or above-average intelligence levels as tested with the Raven Progressive Matrices (Raven and Raven, 2003). All were recruited through non-profit autism organizations, diagnostic centers, autism coaches, and *via* flyers, distributed through social media. The other 13 participants were neurotypical individuals, seven women and six men with an age range from 18 to 25 years (*M* = 23.14, *SD* = 3.76). None of these persons reported use of medication, neurological, or developmental disorders. All participants signed a written informed consent after being informed of the details of the study. They all received 20 euros in exchange for their participation. This study was approved by the medical ethical committee of the University Hospital Brussels, in line with the guidelines of the Declaration of Helsinki (2013).

### Material

In order to test sequence generation, the picture and verbal sequencing tasks were used, including practice trials and their respective non-sequential control task as described in Heleven et al. (2019). In this task, participants see scenarios consisting of four cartoon-like pictures or short sentences that represent four distinct types of sequences: (nonsocial) mechanical events, social scripts, true beliefs, or false beliefs (for an example of a false belief in the picture sequencing task, see Figure 1). All trials were presented in a random order across all sequence types, and the tasks started with a few practice trials to familiarize participants with the instructions and the tasks. We used an extended version of these tasks with an increased number of trials (21 and 25 trials per sequence type for the picture and verbal sequencing task respectively) to increase statistical power and to provide a shorter equivalent in Dutch, but several language versions (Dutch, French, Italian, and English) are available on request.

### Procedure

For this study, we borrowed participants from other studies in which they performed the extended version of the p and verbal sequencing task. We only discuss aspects of these studies that are relevant to the current investigation. Other details can be found in the original papers mentioned below.

Fourteen persons with an ASD diagnosis participated in a training study that investigated the effect of a narrative sequencing and mentalizing training in adults with ASD (Bylemans et al., 2022). Specifically, they investigated training effects on the picture and verbal sequencing task. The tasks were split into two halves. Participants performed half of the tasks before, and the other half after the training. For the current comparison, we only used pre-training data, consisting of half of the material of the picture and verbal sequencing task. Since no manipulation took place at this point, except those inherent to the sequencing tasks, we expect task performances to be unaffected by any aspects of the training study.

**Table 1:** Mean accuracies and response times per task, group, and sequence type.

**Picture task—Accuracies per condition**										
	**False belief**		**True belief**		**Social script**		**Mechanical**		**Control**	
ASD	5.66	(0.44)	5.90	(0.20)	5.81	(0.25)	5.76	(0.27)	5.53	(0.48)
Neurotypical	5.65	(0.50)	5.52	(0.65)	5.88	(0.17)	5.64	(0.33)	5.72	(0.40)
**Verbal task—Accuracies per condition**										
	**False belief**		**True belief**		**Social script**		**Mechanical**		**Control**	
ASD	5.63	(0.50)	5.44	(0.43)	5.87	(0.18)	5.80	(0.18)	5.49	(0.52)
Neurotypical	5.55	(0.46)	5.65	(0.30)	5.83	(0.18)	5.72	(0.35)	5.45	(0.54)
**Picture task—RTtotal per condition**										
	False belief *		True belief *		Social script *		**Mechanical**		Control *	
ASD	28.14	(12.10)	29.00	(12.08)	23.23	(8.04)	12.23	(5.24)	13.63	(3.12)
Neurotypical	20.90	(5.22)	21.82	(4.69)	17.64	(2.27)	17.10	(4.44)	11.16	(2.73)
**Verbal task—RTtotal per condition**										
	**False belief**		**True belief**		**Social script**		**Mechanical**		**Control**	
ASD	32.71	(4.97)	31.61	(3.46)	26.72	(7.34)	27.56	(5.03)	26.40	(10.08)
Neurotypical	33.27	(8.88)	33.02	(9.24)	23.07	(6.39)	28.39	(7.77)	22.61	(6.31)
**Picture task—RTfirst per condition**										
	False belief *		True belief *		Social script *		**Mechanical**			
ASD	18.00	(8.07)	15.53	(3.82)	13.69	(5.96)	11.19	(4.51)		
Neurotypical	13.08	(4.23)	12.85	(3.08)	9.83	(1.50)	9.10	(2.97)		
**Verbal task—RTfirst per condition**										
	**False belief**		**True belief**		**Social script**		**Mechanical**			
ASD	23.75	(10.96)	19.40	(3.23)	16.02	(5.06)	17.63)	(6.34)		
Neurotypical	19.48	(5.15)	18.88	(5.38)	12.38	(3.71)	16.22	(3.85)		

Note. Accuracy totals 6 points for each trial and is determined by 2 points for a correct first and last position, and 1 point for the other intermediate positions, analogous to the scoring system of previous research (Heleven et al., 2019). Numbers between parentheses are standard deviations. Asterisks indicate significant differences between ASD and Neurotypical controls using a one-side t-test with * = *p* < 0.05. RTtotal, Response time from the start of the trial until the last picture was chosen for correct trials only; RTfirst, Response time from the start of the trial until the first picture was chosen. Note that there is no RTfirst for the non-sequencing task that functions as a control condition since no picture selection took place.

Data of the 13 neurotypical individuals were acquired at the Vrije Universiteit Brussel for a noninvasive neurostimulation study. The tasks were split into two halves for this study as well, so it could be used in two separate sessions. Participants performed half of the tasks in one session after receiving neurostimulation, and half of the tasks in the other session after a sham stimulation, which is an inactive stimulation that should not have any effect. For the current comparison, we used data gathered after a sham stimulation only, and only selected persons that had not yet participated in a session in which they received real neurostimulation to exclude potential stimulation or learning effects.

All participants with ASD and three participants without ASD performed the task on a Microsoft Surface Pro 3 Tablet with an attached keyboard, 12" Full HD display (2,160 × 1,440). The other participants performed the task on an HP ZBook laptop with a 15"6 display (1,920 × 1,080). The task ran on E-prime 3 software (Psychology Software Tools, Pittsburgh, PA). The selected halves of the picture and verbal version of the tasks were completed in a counterbalanced order across participants. Each version was conducted following the same procedure. First, participants performed the non-sequential task that functions as a control condition. In each trial, an event was expressed in four cartoon-like pictures or sentences, aligned in the correct chronological order. Participants had to answer a factual question about the events, presented at the bottom of the screen. They could respond yes or no to this question at their own pace by pressing keys 1 or 2 with their right index or middle finger respectively. Second, they carried out the experimental sequencing conditions in which four pictures or sentences were presented in random order. Participants were instructed to line up the pictures/sentences in the correct chronological order by pressing keys 1–4 to indicate the related picture/sentence to be added next in the sequence, using their index to the little finger of their right hand. After the third picture/sentence was selected, participants could restart the trial if they had made a mistake, or confirm their chosen order and continue to the next trial. Participants were asked to respond as accurately and as fast as possible.

### Analysis

We performed the analysis using IBM SPSS Statistics (Version 26). We calculated for each participant and per condition the mean accuracy rates, response time until participants choose the first picture/sentence (RTfirst), and total response times until participants choose the last picture/sentence, omitting erroneous trials (RTtot) per participant and per condition, for the Verbal and Picture Sequencing task separately. Like previous studies, we reasoned that RTfirst, or the time until the first selection, reflects the most critical process of solving the correct sequence, while any additional time might mostly involve processes related to remembering, executing, and updating the sequence (Heleven et al., 2019).

We analyzed these data using a repeated measures ANOVA with Sequence Type (mechanical vs. social script vs. true belief vs. false belief vs. non-sequential control) as a within-participant factor and Group (Neurotypical Controls and Autistic) as a between-participant factor. For analyzing RTfirst, due to the absence of a selection in the control condition, we only included the first four levels of Sequence Type. One-sided independent sample t-tests were computed to further investigate group differences for each Sequence Type separately when the ANOVA showed significant Group effects. Specifically, we expected diminished performance for persons with ASD as compared to the control participants. We anticipate these effects to be most pronounced for processing sequences that are social and require mentalizing, since ASD is particularly linked to problems in social processing (Howlin and Moss, 2012).

## Results

Mean accuracies and response times are listed in Table 1. As expected, accuracy rates for the picture and verbal sequencing tests were at the ceiling, showing no relevant effects.

To support our hypotheses that action sequence generation is impaired for persons with ASD vs. neurotypical controls, the ANOVA should reveal a main effect for Group. For persons with ASD who mainly experience problems in social functioning, we further hypothesized longer response times for social sequences involving mentalizing (and perhaps social scripts), and this differential impairment should be revealed by a significant Group × Sequence Type interaction.

**Figure 2:**
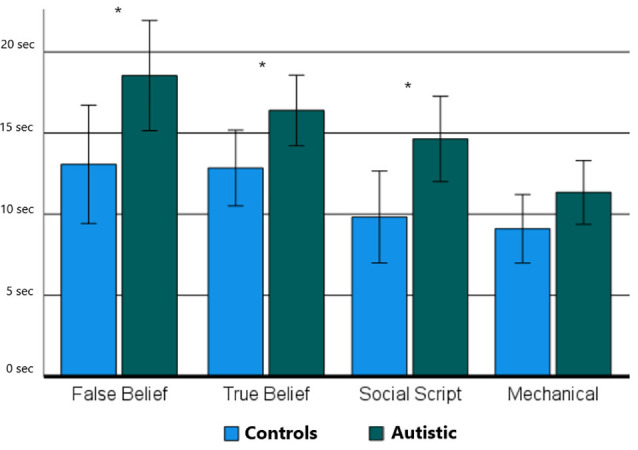
The mean response times until the first response is given per sequence Type (False Belief, True Belief, Social Script, and Mechanical) and Group (Neurotypical Controls and Autistic). Asterisks indicate significant differences between groups as demonstrated using a one-sided independent sample t-test with *p < 0.05.

For the picture sequencing task, an ANOVA on the response times showed a main effect for Group (RTtot: *F*_(1,23)_ = 4.72, *p* = 0.040, $\eta_{p}^{2}$ = 0.16; RTfirst: *F*_(1,23)_ = 4.79, *p* = 0.038, $\eta_{p}^{2}$ = 0.16), and Sequence Type (RTtot: *F*_(1,4)_ = 42.06, *p* < 0.001, $\eta_{p}^{2}$ = 0.63; RTfirst: *F*_(1,3)_ = 19.38, *p* < 0.001, $\eta_{p}^{2}$ = 0.44). No interaction effect between Group and Sequence Type was revealed (RTtot: *F*_(1,4)_ = 2.34, *p* = 0.060, $\eta_{p}^{2}$ = 0.09; RTfirst: *F*_(1,3)_ = 1.33, *p* = 0.272, $\eta_{p}^{2}$ = 0.05). One-sided independent sample t-tests showed significant group differences for all social sequence types (RTtot: *t*_(24)_ = 2.04–2.50, *p* < 0.012–0.028; RTfirst: *t*_(24)_ = 2.01–2.35, *p* < 0.017–0.029), but not for non-social mechanical sequences (RTtot: *t*_(24)_ = 1.14, *p* = 0.134; RTfirst: *t*_(24)_ = 1.40, *p* = 0.086). A similar pattern of significance was revealed after correction for multiple comparisons using Benjamini Hochberg corrections (Benjamini and Hochberg, 1995) with a false discovery rate (FDR) of 5%. Figure 2 shows this pattern of results for the RTfirst data which is very similar to that of the RTtot data.

For the verbal sequencing task, we only observed a main effect of Sequence Type (RTtot: *F*_(1,4)_ = 20.18, *p* < 0.001, $\eta_{p}^{2}$ = 0.48; RTfirst*F*_(1,44)_ = 16.43, *p* < 0.001, $\eta_{p}^{2}$ = 0.41). No significance was shown for a Group maine ffect (RTtot: *F*_(1,23)_ = 0.16, *p* = 0.693, $\eta_{p}^{2}$ = 0.01; RTfirst: *F*_(1,23)_ = 1.22, *p* = 0.280, $\eta_{p}^{2}$ = 0.05) or for an interaction between Group and Sequencing Type (RTtot: *F*_(1,4)_ = 2.08, *p* = 0.089, $\eta_{p}^{2}$ = 0.08; RTfirst: *F*_(1,3)_ = 1.24, *p* = 0.303, $\eta_{p}^{2}$ = 0.05).

## Discussion

This study investigated action sequence generation of high-functioning autistic adults, by comparing their behavioral performances with neurotypical control participants on the picture and verbal sequencing tasks. These tasks recruit the posterior cerebellum (Heleven et al., 2019) and require the understanding of high-level social true and false beliefs, social routines, and nonsocial mechanical functionality. We hypothesized that sequence generation would be impaired in autistic adults since sequential information processing is related to the cerebellum (Leggio and Molinari, 2015), and in particular social sequencing since ASD is associated with dysfunctionality in the posterior cerebellum involved in social mentalizing (D’Mello et al., 2015; Olivito et al., 2018; Velikonja et al., 2019; Van Overwalle et al., 2021).

As expected, results on the picture sequencing task show impaired sequence generation in autistic adults as compared to neurotypical controls for both types of belief sequences and social scripts, but not for mechanical sequences. Our results are in line with the well-established finding that persons with autism mainly have problems linked to mentalizing (Sommer et al., 2018), and have reduced generalized knowledge of what happens in everyday social situations (Loth et al., 2008). However, the observed impairments in generating social, but not mechanical stories of the picture sequencing task provide new evidence for social problems in ASD related to the sequencing of social actions, which has hereto been largely neglected. Note that these differential effects depending on sequence type were only revealed based on direct comparisons between groups per sequence type. Since no interaction effect was revealed, these type-depending effects should be interpreted with caution and further investigated.

The current study’s rationale is based on neuroscientific evidence, but we only investigated behavioral data and therefore cannot draw any conclusions on underlying neurological explanations. We can merely speculate that diminished social sequence processing in autistic adults as compared to neurotypical participants, on the picture sequencing task, is a reflection of cerebellar abnormalities in Crus I and II, regions strongly associated with social sequence processing (Van Overwalle et al., 2020a). Future neuroscientific studies can investigate whether this neural explanation is plausible by the direct comparison of functional brain activation for persons with and without ASD while solving the sequencing task. These studies should take into account the whole brain, including not only cerebellar but also cortical areas that are more traditionally linked to social processing in order to evaluate whether diminished task performance is related to problems with social sequence generation or to more general problems related to mentalizing and social cognition.

An alternative explanation for this impaired performance on the picture sequence task for persons with ASD as compared to neurotypical controls, is a heightened focus on detail in ASD (Gerland, 2003). Participants with ASD might have been distracted by details in the pictures, leading to longer processing times for the pictures. However, this explanation is unlikely since comprehension of most stories relies on details in the pictures. Future studies can further investigate this alternative explanation for example by using eye tracking devices or investigate the effects of training in visual information processing on the task.

Unexpectedly, the verbal sequencing task did not reveal any performance differences between both groups. We speculate that this verbal task might not necessarily require mentalizing to achieve high performance. In general, autistic people are very good at detecting rules and patterns, a cognitive style known as systemizing (Baron-Cohen et al., 2003). This means that their preferred processing style is sometimes rule-based such as in deductive processing in the format of “if p then q”. Given that the sentences contain temporal reference information that marks transitions between actions (e.g., “next”, “after that”, etc.), participants could have used this information to build a logical sequence, therefore avoiding the need to mentalize. Future studies can investigate this explanation further, for example by using neuro-imaging techniques such as fMRI which might reveal differential neural activation for ASD and neurotypical persons while recruiting different mental processes during the performance of the verbal sequence task.

An important limitation of this study is that we borrowed data from other studies, which were not specifically designed to perform the comparisons as discussed in the current study. Due to the procedures followed in these studies, we could only take into account half of the extended versions of the picture and verbal sequencing tasks—although this limitation is not very problematic since the number of trials is still higher than the original sequencing tasks (Heleven et al., 2019; Van Overwalle et al., 2019b). In addition, to limit variability from unwanted sources, we matched the selected participants from both groups on gender and age. However, this resulted in the inclusion of a relatively low number of participants in the current study and did not allow us to avoid all potential variability e.g., due to screen sizes. Our recommendation for future research is to conduct a study which directly compares sequence processing in autistic and neurotypical individuals using the same procedure for both groups. The inclusion of more participants and stimulus material in such a study will most likely lead to increased statistical power and stronger group differences.

Already many valuable assessment tools and tasks have been designed to investigate and diagnose ASD (Janeslätt, 2012), but most were developed for children or adolescents and do not require higher-level social processing. Since the picture and verbal sequencing task can be used in adults and require mentalizing, future studies on sequence processing in ASD can further explore task performance in more detail with the long-term goal of developing new tools for clinical application.

## Conclusion

This study is a first step to systematically investigate sequencing in persons with ASD, including high-level social sequencing that requires mentalizing. Our results confirm that pictorial social sequence generation is impaired in ASD as compared to neurotypical individuals. This is in line with earlier research revealing social (mentalizing) sequence processing to involve the posterior cerebellum and relating ASD to deficits in this cerebellar region. Our results underline the importance of taking into account the cerebellum and its sequence processing function when investigating ASD difficulties. However, more research is needed to confirm the present results.

## Data Availability Statement

The raw data supporting the conclusions of this article will be made available by the authors, upon reasonable request to the corresponding author.

## Ethics Statement

The studies involving human participants were reviewed and approved by the medical ethical committee of the University Hospital Brussels, Vrije Universiteit Brussel. The patients/participants provided their written informed consent to participate in this study.

## Author Contributions

EH, QM, and TB contributed to the study conception and design. Material preparation, data collection and analysis were performed by EH with help from TB and QM. The first draft of the manuscript was prepared by EH and all authors gave their valuable comments and suggestions on the manuscript. All authors contributed to the article and approved the submitted version.

## Funding

This study was funded by the Spearheaded Research Program (SRP57) of the Vrije Universiteit Brussel, Belgium, awarded to Frank Van Overwalle.
